# Relationship between ABO Blood Group Distribution and COVID-19 Infection in Patients Admitted to the ICU: A Multicenter Observational Spanish Study

**DOI:** 10.3390/jcm11113042

**Published:** 2022-05-28

**Authors:** Carlos Jericó, Saioa Zalba-Marcos, Manuel Quintana-Díaz, Olga López-Villar, Iván Santolalla-Arnedo, Ane Abad-Motos, María Jesús Laso-Morales, Esther Sancho, Maricel Subirà, Eva Bassas, Regina Ruiz de Viñaspre-Hernández, Raúl Juárez-Vela, José Antonio García-Erce

**Affiliations:** 1 Internal Medicine Department, Complex Hospitalari Moisés Broggi, 08970 Sant Joan Despí, Spain; cjericoalba@gmail.com; 2 Haematology Department, Hospital Universitario de Navarra, 31008 Pamplona, Spain; saioa.zalba.marcos@navarra.es; 3 PBM Research Group, Idi-Paz Research Institute, 28046 Madrid, Spain; mquintanadiaz@gmail.com (M.Q.-D.); jagarciaerce@gmail.com (J.A.G.-E.); 4 Intensive Care Unit, Hospital Universitario La Paz, 28046 Madrid, Spain; 5 Transfusion Service, Haematology Department, Hospital Universitario de Salamanca, 37007 Salamanca, Spain; olgalopez@usal.es; 6 Department of Nursing-GRUPAC, University of La Rioja, La Rioja, 26006 Logroño, Spain; ivan.santolalla@unirioja.es; 7 Anaesthesia Department, Hospital Universitario Infanta Leonor, 28031 Madrid, Spain; aneabad@hotmail.com; 8 Anaesthesia Department, Corporació Sanitària Parc Taulí, 08208 Sabadell, Spain; mlaso@tauli.cat; 9 Haematology Department, Hospital General de Granollers, 08402 Granollers, Spain; esanchoponce@gmail.com; 10 Haematology Department, Hospital Sagrat Cor, 08029 Barcelona, Spain; msubira@bst.cat; 11 Anaesthesia Department, Complex Hospitalari Moisés Broggi, 08970 Sant Joan Despí, Spain; ebassasp@gmail.com; 12 Blood and Tissue Bank of Navarra, Servicio Navarro de Salud-Osasunbidea, 31003 Pamplona, Spain

**Keywords:** ABO blood-group system, coronavirus infections, multivariate analysis

## Abstract

Since the beginning of the COVID-19 pandemic in December 2019, a relationship between the ABO blood group type and the novel coronavirus SARS-CoV-2, the etiological agent of COVID-19, has been reported, noting that individuals with the O blood group are the least likely to be infected. Spain is one of the most badly affected countries worldwide, with high rates of patients diagnosed, hospitalized, and deceased due to COVID-19 infection. The present study aimed to analyze the possible relationship of ABO in COVID-19 patients hospitalized in different Spanish centers during the first wave of the COVID-19 pandemic, for which the ABO group was available. Physicians from the transfusion services of different Spanish hospitals, who have developed a multicenter retrospective observational study, were invited to participate voluntarily in the research and 12,115 patients with COVID-19 infection were admitted to the nine participating hospitals. The blood group was known in 1399 cases (11.5%), of which 365 (26.1%) were admitted to the ICU. Regarding the distribution of ABO blood groups, a significant increase in the non-O blood groups and reduction for the O blood group was observed in patients hospitalized due to COVID-19, compared to the reference general population. Among the patients admitted to the ICU, after multivariate analysis, adjusted for the rest of the confounding variables, patients with the O blood group presented a significantly lower risk for admission to the ICU. We conclude that an association was observed between patients with the O blood group and their lower susceptibility to SARS-CoV-2 infection, both for those admitted to the hospitalization ward and for those who required admission to the ICU.

## 1. Introduction

Since the description in Wuhan, China, in December 2019, of the first cases of pneumonia caused by a novel coronavirus SARS-CoV-2, the so-called new coronavirus infection 2019 (COVID-19) [1] has spread quickly to most countries in the world, leading to the declaration of a pandemic by the World Health Organization. Spain is one of the most affected countries worldwide, with high rates of patients diagnosed, hospitalized, and deceased due to COVID-19 infection [2]. Different characteristics such as advanced age, male sex or some chronic diseases seem to increase the risk of more severe disease due to SARS-CoV-2 [3,4,5,6]. Landsteiner ABO blood groups were described on the basis of carbohydrate epitopes, which are present on the surface of erythrocytes and in many other types of human cells [7]. The presence of A and B antigens has been associated with increased susceptibility to the development of infections, cardiovascular diseases, and cancer [8]. Since the beginning of the COVID-19 pandemic, several publications have reported that there was a relationship between ABO blood group type and SARS-CoV-2 infection, noting that individuals with the O blood group are least likely to be infected [9,10,11,12,13,14,15]. This association between blood group and coronavirus infection had already been observed in SARS infection in 2005, where individuals with the O blood group were less likely to be infected by the virus [16]. ABO blood groups present differences in their distribution worldwide. This study investigates the possible relationship between the ABO group and susceptibility to the disease in different Spanish centers during the first wave of the COVID-19 pandemic, for which the ABO group was available, and compares the results obtained with those reported in recent months from cohorts of COVID-19 patients from different areas of the world.

## 2. Experimental Section

### 2.1. Study Design and Eligibility Criteria

This multi-center retrospective observational study included data from patients who were admitted to hospitals because of a SARS-CoV-2 infection. Only data from patients admitted to the ICU with a definite SARS-CoV-2 diagnosis using reverse transcription-polymerase chain reaction were included. Physicians from the transfusion services or related to the transfusion committees of different Spanish hospitals (Hospital Moisès Broggi, Hospital Complex of Navarra, Hospital Garcia Orcoyen of Estella, University Hospital of La Paz, Hospital Infanta Leonor, Hospital Sagrat Cor, Hospital of Parc Tauli, Hospital University of Salamanca, and Hospital of Granollers) were invited to participate voluntarily in the study. The eligibility criteria for inclusion were: patients over 18 years of age, confirmed SARS-CoV-2 infection that required hospital admission for at least 24 h, and known ABO blood group type, either because of a request for blood transfusion during admission for COVID-19 infection, or due to a prior determination of the patient’s blood group that was registered in the medical record.

### 2.2. Data Collection and Confidentiality

Patients admitted to the different participating hospitals between 1 March and 31 May 2020, and who met the inclusion criteria, were selected. Data obtained from the review of medical records were anonymized by assigning a numerical code for each patient and center, and were subsequently included in a common database.

The following data were recorded: type of blood group (A, B, AB, O), age, sex, comorbidities (hypertension, diabetes, obesity-body mass index ≥ 30 kg/m^2^, cardiovascular disease, obstructive lung disease, malignancy), laboratory tests (lymphocyte absolute count, C-reactive protein, D-dimer) and complications (ICU—Intensive Care Unit—admission, thrombotic complications, and mortality).

ABO Blood group distribution data in the general population not infected by COVID-19 was extracted from the donor and transfusion management application e-Delphyn BB version 8.0.17.2 (Hemasoft^®^, Valladolid, Spain) of the Blood and Tissue Bank of Navarra, Servicio Navarro de Salud-Osasunbidea, Pamplona, Spain.

All procedures were performed in accordance with legal provisions of the protection of personal data in Spain (LOPD 3/2018) and European Union Regulations (EU) 2016/6799, on the physical protection of the treatment of personal data. The study was compliant with the Declaration of Helsinki. The Research Ethical Committee of the Bellvitge University Hospital approved the study (Reference: PR176 20-CSI 20 69-), including the exemption of the requirement for informed consent. 

### 2.3. Statistical Analysis

Due to the observational nature of the study, the sample size was not calculated, defined as the number of patients who fulfilled the inclusion criteria. Demographic and clinical data were described by frequencies and percentages (%) for categorical variables and using the mean ± standard deviation (SD) or median ± interquartile range (IQR) for quantitative variables. The baseline parameters were compared between groups using the nonparametric Mann-Whitney test or the parametric Student’s t-test for continuous variables, and using the chi-square test for categorical variables. For the analysis of the evolution of the control parameters and complications, the trend test for proportions, the linear trend test or the Kruskal-Wallis test were used. A *p* value lower than 0.05 was considered statistically significant. The analyses were performed with IBM SPSS v. 25.0 (Armonk, NY, USA).

## 3. Results

**Figure 1:**
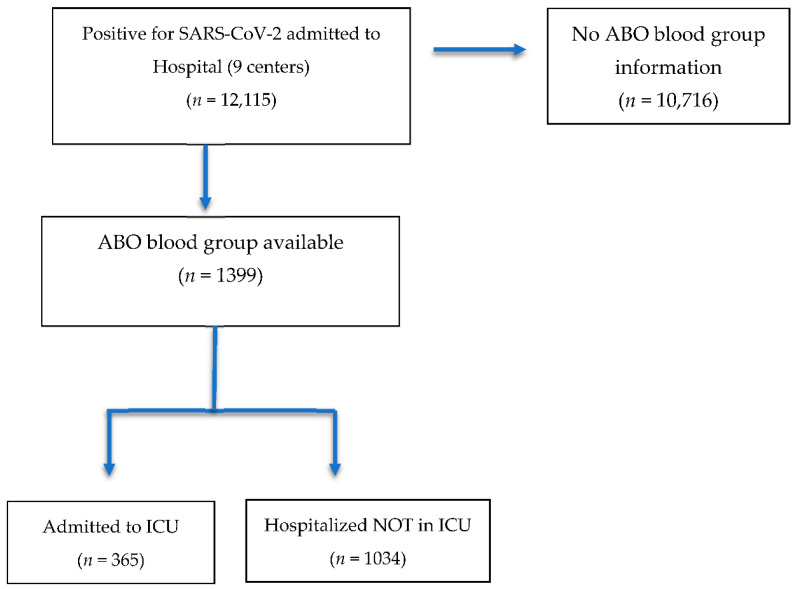
Flow chart of selected cases.

12,115 patients with SARS-CoV-2 were admitted to the nine participating hospitals between March 1 and May 31. Of all patients, the blood group was known in 1399 cases (11.5%), of which 365 (26.1%) were admitted to the ICU (Figure 1). 

Because of the descriptive results for the 1034 patients not admitted to the ICU, it is observed that men are predominant at 52.9% compared to 47.1% of women, and with a mean age of 81 years. The average number of lymphocytes is 0.80 × 10^6^/L; C-reactive protein is 101.7 mg/L and D-dimer is 1392.5 ng/mL. Regarding blood groups, there is a predominance of groups A (46.2%) and O (43%) compared to a lower percentage of groups B (7.3%) and AB (3.5%). Likewise, based on the information available on comorbidities, it can be seen that 67.6% are hypertensive, 30.3% are diabetic, 32% are obese, 39.1% have previous cardiovascular disease, 19.2% have pulmonary obstruction and 20.1% have a neoplastic disease (malignant). On the other hand, there are only 3.1% with thrombotic complications, and a mortality outcome of 35.1% with a positive exitus. Concerning the 365 patients admitted to the ICU, there is also a predominance of men at 71.2% compared to 28.8% women. The mean age of patients admitted to the ICU is lower at 66 years. The average of lymphocytes is 0.47 × 10^6^/L; C-reactive protein is 255.4 mg/L and D-dimer is 9039 ng/mL. Regarding the blood groups of patients admitted to the ICU, there is a predominance of groups A (46.6%) and O (38.9%) compared to a lower percentage of groups B (10.7%) and AB (3.8%). 51% are hypertensive, 24.9% diabetic, 34.2% obese, 15.9% have previous cardiovascular disease, 17.5% pulmonary obstruction and 13.7% a neoplastic disease (malignant). In addition, 21.1% had thrombotic complications and there was a mortality outcome of 43% with a positive exitus (Table 1). 

Regarding the distribution of ABO blood groups, there is a significant increase in the percentage of those from group O compared to the reference general population (Table 2 and Table 3).

When comparing the distribution of ABO groups, we observed the existence of statistical differences between the distribution of blood groups among the general population and the group of patients admitted to the hospital for COVID, non-ICU patients (Chi-square 12.4, df = 3, *p* < 0.01) and the group of patients admitted to the ICU (Chi-square 20.02, gl = 3, *p* < 0.01). People admitted to the hospital for COVID are more likely to have group B and AB blood than group A when compared to the general population and a statistically significantly lower chance that their blood is group O than group A (Table 1). If we compare people admitted to the ICU for COVID-19 with the general population, this trend persists with statistically significant differences (Table 2).

When comparing the blood group between people admitted to the hospital and those who need admission to the ICU, people who are admitted to the ICU for COVID-19 are more likely to have blood group AB or B than group A, and a less statistically significant probability of having the O group (Table 4 and Table 5).

## 4. Discussion

In this study, an association was observed between patients with the O blood group and their lower susceptibility to SARS-CoV-2 infection, both for those admitted to the hospitalization ward and for those who required admission to the ICU. This finding of a possible protective effect of the O blood group in patients with COVID-19 infection requiring conventional hospitalization has already been observed from the initial description in the Chinese population of Wuhan, and later in other territories with a high spread of COVID-19 infection, in North America, the Middle East, and Europe [9,10,16,17,18,19]. In addition, the lower presence of patients with blood group O among those infected by COVID compared to reference populations has also been observed in convalescent plasma donors and even in population studies such as those recently published in Canada and Denmark, with similar results, despite the marked differences in the distribution of ABO blood groups in both populations [20,21,22] 

In Spain, the association of blood group O with a lower risk of COVID-19 infection was described in some Spanish cohorts included in a genome study [12], while no association of any blood group with COVID infection was observed in a study of hospitalized patients for SARS-CoV-2 infection when all had been transfused during admission. However, the association also found in this study between blood group O and a lower probability of admission to the ICU, which is maintained after adjustment for possible confounding factors, had not been previously described in the published studies that included the analysis of the blood group in critically ill patients, except in the study conducted in multiple US ICUs, although only for non-Hispanic white participants [21,22,23,24,25]. In our study, the distribution of patients by race was not collected, but perhaps a bias should be suggested because the patients admitted to the ICU in the first wave in Spain were younger, probably due to the presence of a higher percentage of migrants [26], mainly of Hispanic race, in which the highest prevalence of blood group O is well known. Regarding mortality, no relationship was observed with the ABO blood group in the present series, while there was a greater association between blood group A and mortality and a lower among carriers of blood group O, both in the study by Zhao in Wuhan, and in the part of the multicenter Spanish study by Muñiz et al., in which patients hospitalized for COVID infection who required a blood transfusion during admission were included [9,27]. Among patients with thrombotic complications, no association with the ABO blood group was observed either. Only in a preliminary study, carried out in one of the centers participating in this study, was there a higher prevalence of thrombotic complications among patients with blood group B, although it must be considered that there were very few patients included with blood group B. Natural anti-A or anti-B antibodies could bind to the viral S protein and block its interaction with ACE2, providing protection by blocking the interaction between the virus and its receptor.

Subjects in group O have a lower-than-average factor VIII and VWF titer, which may offer protection against associated complications. In both situations, blood group O appears protective in comparison with non-O-types. The protective effect on infection could be mediated either by natural anti-A and anti-B antibodies or by a lower efficiency of furin cleavage in the O blood group individuals [15,28,29,30,31].

## 5. Conclusions

In this observational study, an association was observed between patients with the O blood group and their lower susceptibility to SARS-CoV-2 infection, both for those admitted to the hospitalization ward and for those who required admission to the ICU. The present study has some limitations associated with a retrospective observational analysis of a multicenter cohort.

## 6. Limitation

The present study has the limitations of many observational studies. We cannot establish cause-effect relationships. On the other hand, the most common blood group in Spain is the O group, which could lead to overrepresentation.

On the other hand, the criterion for admission to the ICU is a variable that researchers have not been able to control, as it is a clinical criterion; however, in our country the ABO group was not a criterion for admission to the ICU.

## Figures and Tables

**Table 1:** Characteristics of patients with COVID-19 infection.

	Patients No-ICU (*n* = 1034)	Patients ICU (*n* = 365)
Sex, *n* (%)		
Male	547 (52.9)	260 (71.2)
Female	487 (47.1)	105 (28.8)
Age (years)	81 (15)	66 (15)
ABO Blood Group, *n* (%)		
A	478 (46.2)	170 (46.6)
B	75 (7.3)	39 (10.7)
AB	36 (3.5)	14 (3.8)
O	445 (43)	142 (38.9)
Comorbidities, *n* (%)		
Hypertension	699 (67.6)	186 (51)
Diabetes	313 (30.3)	91 (24.9)
Obesity (BMI ≥ 30 kg/m^2^)	331 (32)	114 (34.2)
Cardiovascular disease	404 (39.1)	58 (15.9)
Obstructive lung disease	199 (19.2)	64 (17.5)
Malignancy	208 (20.1)	50 (13.7)
Laboratory, median [IQR]		
Lymphocytes (×10^6^/L)	0.80 [0.78]	0.47 [0.40]
C-reactive protein (mg/L)	101.7 [148.9]	255.40 [216.6]
D-dimer (ng/mL)	1392.5 [2238]	9039 [21,010]
Thrombotic complications, *n* (%)	32 (3.1)	77 (21.1)
Mortality, *n* (%)		
Exitus	363 (35.1)	157 (43)

BMI: Body Mass Index; IQR: Interquartile range [Q1–Q3]; ICU: Intensive Care Unit.

**Table 2:** Comparison between ABO group distribution in the group of patients with COVID-19 admitted to the hospital (patients no ICU) and control group: general population * *p* < 0.05.

ABO Blood Group	Patients No ICU *n* (%)	General Population *n*(%)	OR (IC 95%)
O	445 (43.0)	87,605 (48.0)	1 ^†^
A	478 (46.2)	78,402 (43.0)	1.20 (1.05–1.36) *
B	75 (7.3)	11,725 (6.4)	1.26 (0.99–1.60)
AB	36 (3.5)	4652 (2.6)	1.52 (1.08–2.13) *
Total	1034 (100.0)	182,384 (100)	

^†^ Since value 1 is excluded from the range of the interval, we can say that the findings are statistically significant.

**Table 3:** Comparison between ABO group distribution in patients with COVID-19 admitted to the intensive care unit (patients ICU) and control group: general population. * *p* < 0.05.

ABO Blood Group	Patients ICU *n* (%)	General Population *n* (%)	OR (IC 95%)
O	142 (38.9)	87,605 (48.0)	1 ^†^
A	170 (46.6)	78,402 (43.0)	1.33 (1.07–1.67) *
B	39 (10.7)	11,725 (6.4)	2.05 (1.44–2.92) *
AB	14 (3.8)	4652 (2.6)	1.85 (1.07–3.20) *
Totals	365 (100.0)	182,384 (100)	

^†^ Since the value 1 is excluded from the range of the interval, we can say that the findings are statistically significant.

**Table 4:** Comparison between ABO group distribution in patients with COVID-19 admitted to the intensive care unit (patients ICU) and control group: patients with COVID-19 admitted to the hospital (patients no ICU). * *p* < 0.05.

ABO Blood Group	Patients ICU *n* (%)	Patients No ICU *n* (%)	OR (IC 95%)
O	142 (38.9)	445 (43.0)	1 ^†^
A	170 (46.6)	478 (46.2)	1.08 (0.89–1.32)
B	39 (10.7)	75 (7.3)	1.41 (1.05–1.89) *
AB	14 (3.8)	36 (3.5)	1.16 (0.73–1.85)
Totals	365 (100.0)	1034 (100.0)	

^†^ Since the value 1 is excluded from the range of the interval, we can say that the findings are statistically significant. After multivariate analysis, adjusted for the rest of the confounding variables, patients with the O blood group presented a significantly lower risk for admission to the ICU.

**Table 5:** Multivariate analysis: ABO blood type versus ICU admission. The referent is blood type A. We express the results of the significance of the variables necessary in the multivariate analysis, adjusted for the rest of the variables (age, sex, lymphocyte count, D-dimer, and C-reactive levels, diagnoses of hypertension, diabetes, or obesity, and previous cardiovascular, obstructive lung or malignant diseases.) The significance is expressed * *p* ≤ 0.005, *p* < 0.001 **.

ABO Blood Group	Admitted to ICU	Adjusted OR	95% CI	*p*
A	170 (46.6)	-----	-----	0.018
B	39 (10.7)	1.27	[0.55–2.90]	0.577
AB	14 (3.8)	0.60	[0.17–2.08]	0.416
O	142 (38.9)	0.52	[0.33–0.81]	0.004 *
Age		0.91	[0.89–0.93]	<0.001 **
Sex		0.52	[0.33–0.82]	0.005 *
Hypertension		0.74	[0.46–1.18]	0.204
Diabetes		1.17	[0.73–1.85]	0.517
Obesity		1.57	[0.97–2.53]	0.065
Cardiovascular disease		0.38	[0.23–0.64]	<0.001 **
Obstructive lung disease		1.06	[0.63–1.78]	0.825
Malignancy		0.88	[0.68–1.12]	0.298
Lymphocytes		0.33	[0.22–0.50]	<0.001 **
C-reactive protein		1.00	[1.00–1.00]	<0.001 **

Chi-Squared test = 10.020; *p*-value = 0.264 (>0.05); * Sig. (≤0.05). R-squared multiple = 0.426 (43%); R-squared adjusted = 0.591 (59%). ICU: Intensive Care Unit; OR: Odds Ratio; CI: Confidence Interval.

## Data Availability

The anonymized data presented in this study are available at the request of the last author. The data are not publicly available due to current personal data protection legislation.
